# In silico analysis of selected polyphenols as potential multitarget rheumatoid arthritis modifying agents

**DOI:** 10.1007/s11033-026-11440-7

**Published:** 2026-01-16

**Authors:** Shiva Sharma, Sudheesh K. Shukla, Krishna K. Govender, Penny P. Govender

**Affiliations:** 1 Department of Chemical Sciences, University of Johannesburg, Johannesburg, 2028 South Africa; 2 Department of Biosciences, Graphic Era (Deemed to be University), Dehradun, 248002 Uttarakhand India

**Keywords:** Polyphenols, Rheumatoid arthritis, Network pharmacology, Molecular docking, Multitargeting

## Abstract

**Introduction:**

Rheumatoid arthritis (RA) arises from a complex inflammatory network involving cytokines, kinases, and matrix degrading enzymes. Methotrexate is the clinical standard but is limited by poor pharmacokinetics and a narrow mechanism.

**Methodology:**

This study evaluates six polyphenols sinapic acid, catechin, galangin, piceatannol, umbelliferone, and pinocembrin against fifteen validated RA targets and benchmarks them against methotrexate.An integrated in silico framework assessed ADME properties, molecular docking, and network pharmacology. ADME screening included Lipinski compliance, GI absorption, solubility, CYP450, and bioavailability.

**Results:**

All polyphenols fullfill Lipinski’s criteria and showed moderate lipophilicity, high predicted GI absorption, favourable solubility, minimal CYP450 interactions, and bioavailability scores of 0.55. Methotrexate displayed one rule violation, extreme hydrophilicity, poor permeability, low GI absorption, and a bioavailability score of 0.11. Docking confirmed methotrexate as the strongest single-target binder; however, catechin, piceatannol, galangin, and umbelliferone demonstrated strong affinities (–6.1 to –9.9 kcal/mol), particularly to iNOS, COX-2, and MMP-9. Network analysis highlighted modulation of TNF, IL-17, JAK–STAT, NF-κB, RA, and arachidonic-acid pathways and GO linked to inflammation and matrix degradation.

**Conclusion:**

The polyphenols, in combination with catechin, are pharmacokinetically robust multitarget modulators capable of suppressing cytokine signalling, kinase activation, NF-κB transcription, and matrix degradation, therefore positing these phytochemicals as promising for next-generation RA therapeutics.

**Graphical Abstract:**

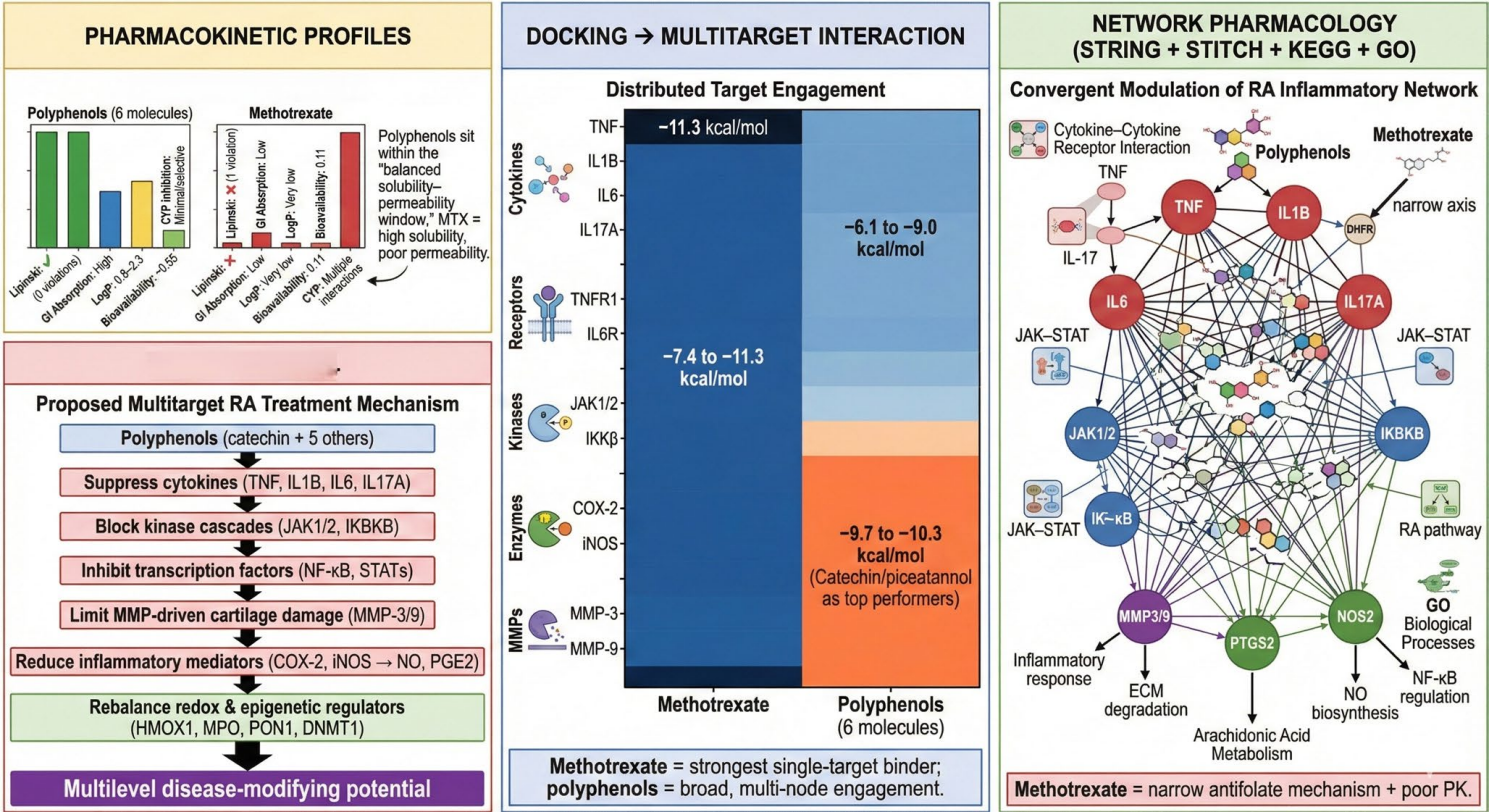

**Supplementary Information:**

The online version contains supplementary material available at 10.1007/s11033-026-11440-7.

## Introduction

Rheumatoid arthritis (RA) is a chronic autoimmune disorder marked by progressive synovial inflammation, cartilage degradation, and bone erosion, culminating in irreversible joint deformities and functional impairment [1]. According to WHO (2019), more than 13 million people experience severe (moderate or severe) Rheumatoid arthritis, 70% of them are women, and 55% are older than 55. RA has a female predominance (3:1) between the fourth and sixth decades of life, varying by ethnicity and geographical location [2]. Rheumatoid Arthritis imposes a socioeconomic burden too, with direct and indirect costs exceeding $19 billion annually in the United States alone [2, 3]. Beyond joints, RA is associated with extra-articular complications, including cardiovascular disease, pulmonary fibrosis, and increased mortality, reducing life expectancy by 3–10 years [3–5]. Pathogenesis driven by dysregulated immune responses involves T cells, B cells, macrophages, and fibroblast-like synoviocytes (FLS). Pro-inflammatory cytokines TNF-α, IL-1β, IL-6, and IL-17 A recruit immune cells, activate osteoclasts, and cause matrix degradation, leading to pannus formation and subsequent joint destruction [3, 5]. RA management involves symptomatic relief using NSAIDs, corticosteroids, and disease-modifying antirheumatic drugs (DMARDs), targeting disease progression. Despite advances, incomplete responses (approximately 40%), adverse effects, overpriced costs, pathway redundancy, and ongoing structural damage need safe, cost-effective, multitargeted therapies that address both inflammation and joint destruction [6]. Natural products show approximately 50% of FDA-approved drugs between 1981 and 2019, including aspirin, morphine, paclitaxel, and artemisinin. In inflammatory diseases, natural products offer multi-target activity for complex pathophysiology, provide structurally diverse bioactive scaffolds, are supported by dietary and ethnopharmacological use, and offer novel pharmacophores. Polyphenols are a large class of plant secondary metabolites (More than 8,000 compounds) with multiple phenolic hydroxyl groups on aromatic rings. Epidemiological evidence tells us that polyphenol-rich diets lead to reduced incidence and severity of RA and other inflammatory diseases [7–9]. Polyphenols target pro-inflammatory signalling cascades and inhibit NF-kB, MAPK, and JAK–STAT pathways, thereby attenuating phosphorylation-driven signal transduction and suppressing inflammatory gene transcription [7]. At cytokine level, polyphenols directly reduce the production of TNF-α, IL-1β, IL-6, and IL-17 A, and enhance production of anti-inflammatory mediators IL-10 and TGF-β, and shifts the immune system from a pro-inflammatory to a resolving phenotype. Polyphenols also suppress COX-2, iNOS, and matrix metalloproteinases, and results in decreased prostaglandin and nitric oxide synthesis, to reduce cartilage and extracellular matrix degradation. Antioxidant activity scavenges reactive oxygen species, chelates, pro-oxidant metal ions, and activates Nrf2/ARE dependent cytoprotective gene expression, to maintain mitochondrial integrity, therefore collectively limiting oxidative amplification of synovial inflammation [5, 7, 9]. Preclinical models of polyphenols (resveratrol, EGCG, quercetin, curcumin, apigenin, kaempferol) reduce synovial inflammation, MMP activity, cytokine production, and cartilage/bone damage in CIA, AIA, and K/BxN, and osteoclastogenesis in arthritis [10–12]. Clinical studies indicate modest improvements in DAS28 and reductions in inflammatory markers using EGCG, curcumin, and pomegranate [13]. However, systematic evaluation against RA-relevant targets is limited [14]. Therefore, this study aims to assess the binding affinity of five merely studied phytoconstituents (sinapic acid, galangin, piceatannol, umbelliferone, pinocembrin), one widely studied (catechin) and one DMARD, Methotrexate, as a control against 15 RA-associated targets. This study will elucidate protein-ligand interactions, PPI, polyphenol-target networks, drug-likeness, prediction of polyphenols with optimal multi-target profiles, and structure-activity relationships for designing next-generation polyphenol therapeutics.

## Methodology

Five merely studied phytoconstituents for RA treatment: Sinapic Acid (637775), Galangin (5281616), Piceatannol (667639), Umbelliferone (5281426), and Pinocembrin (68071) were selected to evaluate their efficacy. Their performance was compared with Catechin (72276), a widely studied phytoconstituent and with the reference DMARD methotrexate as a positive control against a total of 15 selected RA-associated protein targets (Supplementary Table 1), encompassing cytokines, receptors, acute-phase mediators, matrix metalloproteinases, kinases, and inflammatory enzymes. All ligands were subjected to in silico ADME and drug-likeness screening using SwissADME. physicochemical descriptors (molecular weight, TPSA, HBA/HBD, rotatable bonds), lipophilicity (multiple logP models), aqueous solubility (ESOL, Ali, Silicos-IT), gastrointestinal absorption, and blood–brain barrier (BBB) permeability was computed, along with Lipinski, Veber, and related rule-based filters [15]. The BOILED-Egg model was used to visualize passive GI absorption and BBB penetration, and to predict cytochrome P450 inhibition (CYP1A2, CYP2C9, CYP2C19, CYP2D6, CYP3A4) [16]. Compounds meeting at least 80% of pre-defined pharmacokinetic criteria (favorable lipophilicity, high GI absorption, no or minimal rule violations, non-problematic CYP profile).

Protein preprocessing includes removal of crystallographic water molecules (structurally conserved in the active site), deletion of co-crystallized ligands and ions where appropriate, addition of polar hydrogens, and assignment of Kollman or comparable partial charges [17]. Ligand structures were downloaded in SDF format from PubChem, energy-minimized, and converted to PDBQT format with appropriate protonation states at physiological pH. Molecular docking was performed using CB-Dock, which employs CurPocket to detect putative binding cavities automatically and generates tailored docking boxes for each cavity [18], Supplementary Fig. 1. For each ligand–protein pair, the pose with the lowest binding energy was selected. Mapping of ligand–protein interaction would be performed using STITCH platform at a minimum confidence threshold of 0.7. Polyphenol and methotrexate were evaluated for direct binding, activation or inhibition, and combined interaction scores with human proteins, integrating data from experiments, databases, and text-mining. In parallel, protein–protein interaction (PPI) networks was done by STRING for 15 RA-associated targets as seeds and a confidence cutoff of ≥ 0.7. The resulting network will be analyzed to identify hub nodes (based on degree and betweenness centrality), tightly connected signaling clusters and enriched interaction patterns. Network topological features will be correlated with docking affinities and STITCH scores to determine whether polyphenols preferentially engage network hubs in RA pathogenesis [18]. RA target set, with additional high-confidence STITCH-derived targets (confidence ≥ 0.7), was subjected to KEGG pathway analysis using standard over-representation approaches with Benjamini–Hochberg (BH) correction for multiple testing (adjusted *p* < 0.05). Gene Ontology (GO) analyses was performed for Biological Process, Molecular Function, and Cellular Component categories using BH-adjusted *p* < 0.05 as the significance cutoff [18].

## Results

**Fig. 1:**
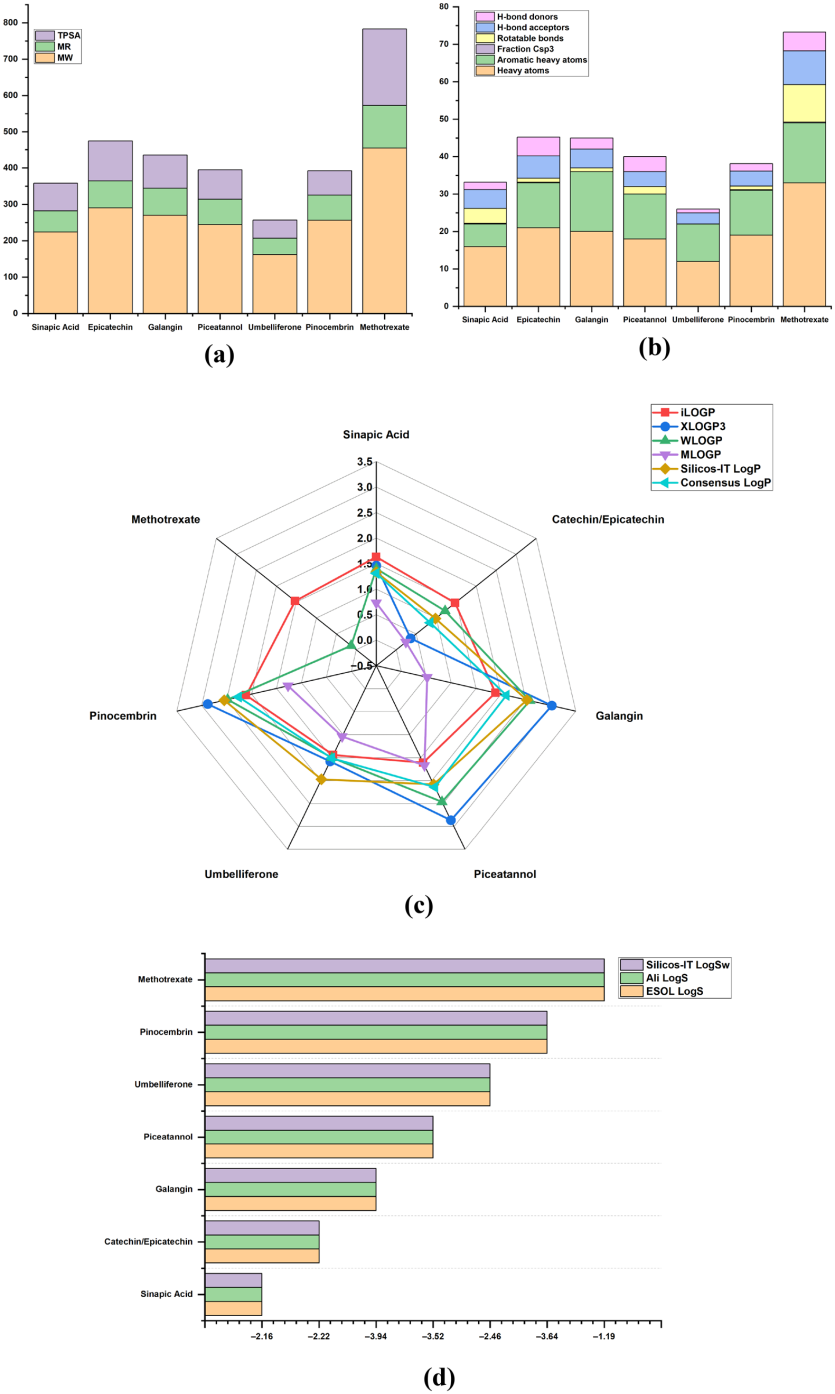

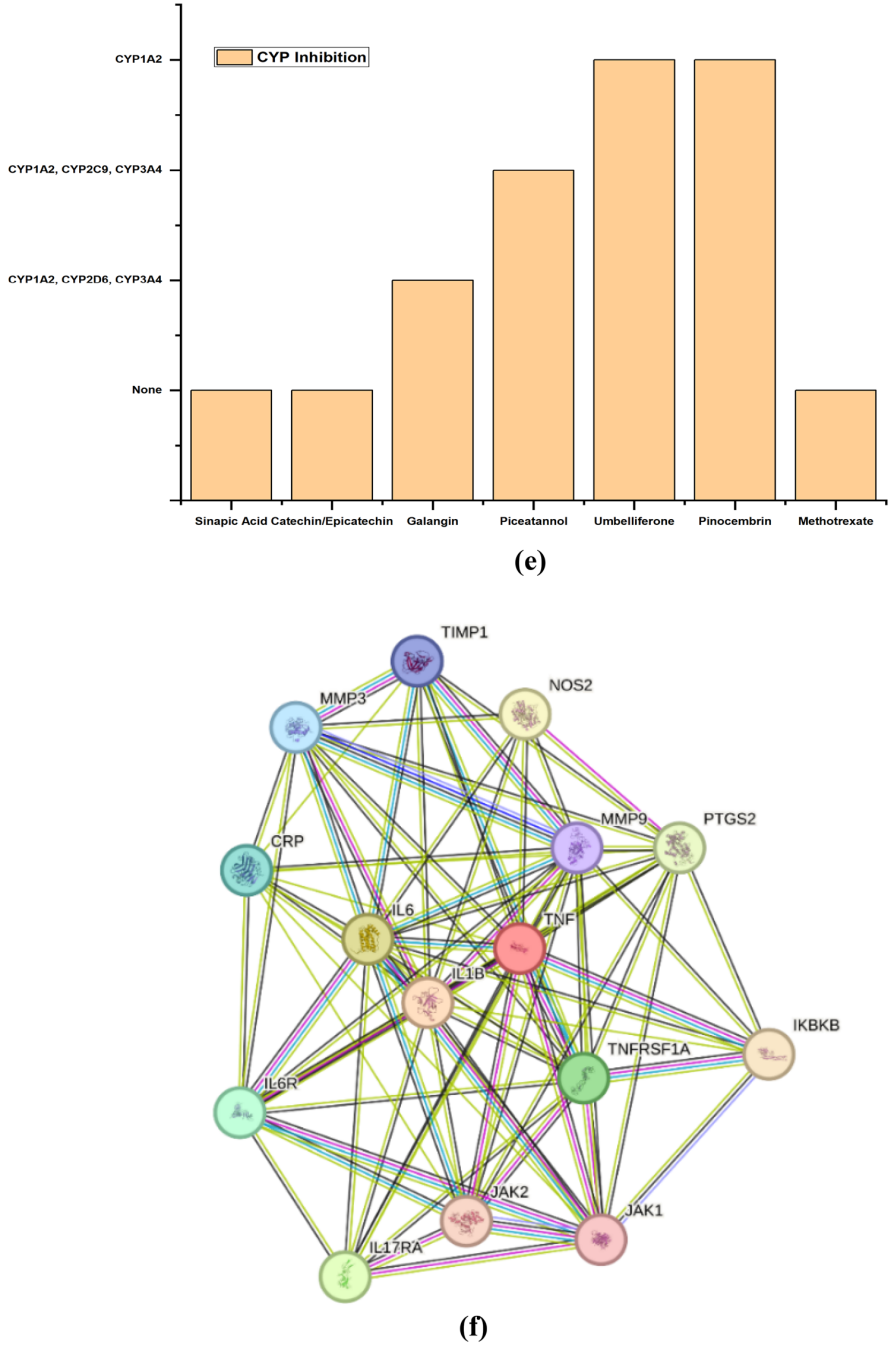
(**a**) Physicochemical Analysis, (**b**) Physicochemical Analysis, (**c**) Lipophilicity, (**d**) Water Solubility. (**e**) CYP Inhibition. (**f**) STRING-PPI Network Analysis

ADME profiling showed that all six polyphenols satisfied Lipinski’s rule of five without violations and indicate optimal MW, TPSA, and hydrogen-bonding, with logKp values between 5.76 and − 7.82 cm/s and uniform bioavailability scores of 0.55–0.56, Fig. 1(a-b). Methotrexate exhibited one Lipinski violation, lower permeability (logKp − 10.39 cm/s), and a bioavailability score of 0.11, consistent with suboptimal oral performance, (Table 1). Radar plot lipophilicity analysis demonstrated that polyphenols clustered at moderateconsensus LogP values (approximately 1–3) across iLOGP, XLOGP3, WLOGP, MLOGP, and Silicos IT models, indicating a favorable solubility–permeability, Fig. 1(c). Whereas methotrexate occupied the low LogP region, reflecting extreme hydrophilicity and poor membrane permeation. Solubility models (ESOL, Ali, Silicos-IT) confirmed that methotrexate is highly soluble but poorly permeant. Whereas, polyphenols, particularly pinocembrin and umbelliferone, exhibited intermediate solubility, compatible with efficient absorption and tissue penetration, Fig. 1(d). Absorption and distribution predictions indicated high GI absorption for all phytomolecules, non-BBB permeability for most compounds, and limited P-gp liability, with only catechin and methotrexate as substrates. Methotrexate combined low GI absorption with P-gp efflux, reinforcing its unfavourable oral pharmacokinetic profile relative to the polyphenols. CYP450 liability remained low across sinapic acid, catechin, and methotrexate showed no predicted CYP inhibition, Fig. 1(e). Galangin and piceatannol inhibited multiple isoforms (CYP1A2, CYP2D6/CYP2C9, CYP3A4) and umbelliferone and pinocembrin selectively inhibited CYP1A2, indicating a manageable, isoform-restricted interaction risk in combination regimens (Table 1). Network-level analysis of 15 RA-associated proteins shows a highly interconnected inflammatory backbone centred on TNF, IL1B, IL6, IL17A, JAK1/JAK2, IKBKB, MMP3/MMP9, PTGS2, and NOS2. High-confidence edges such as TNF–TNFRSF1A, IL6–IL6R, JAK1–JAK2, MMP3/MMP9–TIMP1, and PTGS2–NOS2 highlighted hubs and bottlenecks that integrate cytokine receptors, JAK–STAT, NF-κB, IL-17, TNF, and arachidonic-acid pathways, which directly or indirectly engaged by the polyphenols, Fig. 1(f), Supplementary Table 2.

**Table 1:** Drug-Likeness and ADMET characteristics

Molecule	logKp (cm/s)	Lipinski	Bioavailability score	GI absorption	BBB permeant	*P*-gp substrate	CYP inhibition
Sinapic acid	–6.63	0	0.56	High	No	No	None
Catechin	–7.82	0	0.55	High	No	Yes	None
Galangin	–5.8	0	0.55	High	No	No	CYP1A2, CYP2D6, CYP3A4
Piceatannol	–5.76	0	0.55	High	No	No	CYP1A2, CYP2C9, CYP3A4
Umbelliferone	–6.17	0	0.55	High	Yes	No	CYP1A2
Pinocembrin	–5.82	0	0.55	High	Yes	No	CYP1A2
Methotrexate	–10.39	1	0.11	Low	No	Yes	None

**Fig. 2:**
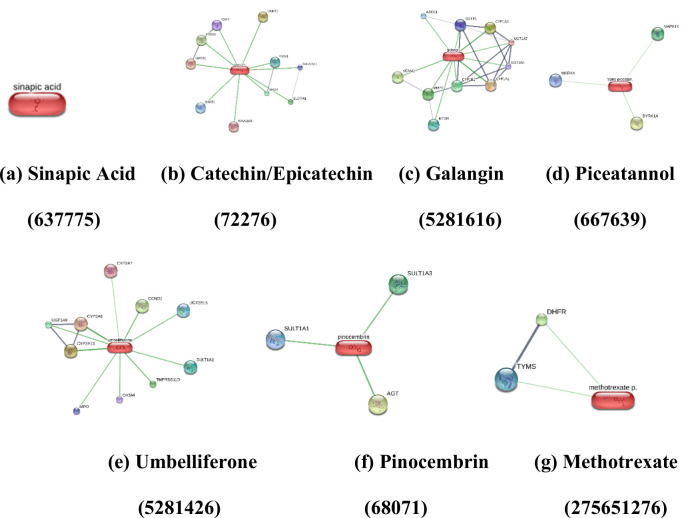
STITCH Chemical–Protein Interaction Mapping

STITCH mapping indicates that catechin, piceatannol, galangin, umbelliferone, and pinocembrin form distinct multi-target interactions (Supplementary Table 3) involving metabolic enzymes, adhesion molecules, oxidative stress mediators, and epigenetic regulators, while methotrexate retains a narrow DHFR–TYMS–centered antifolate profile Fig. 2(a-g).

**Fig. 3:**
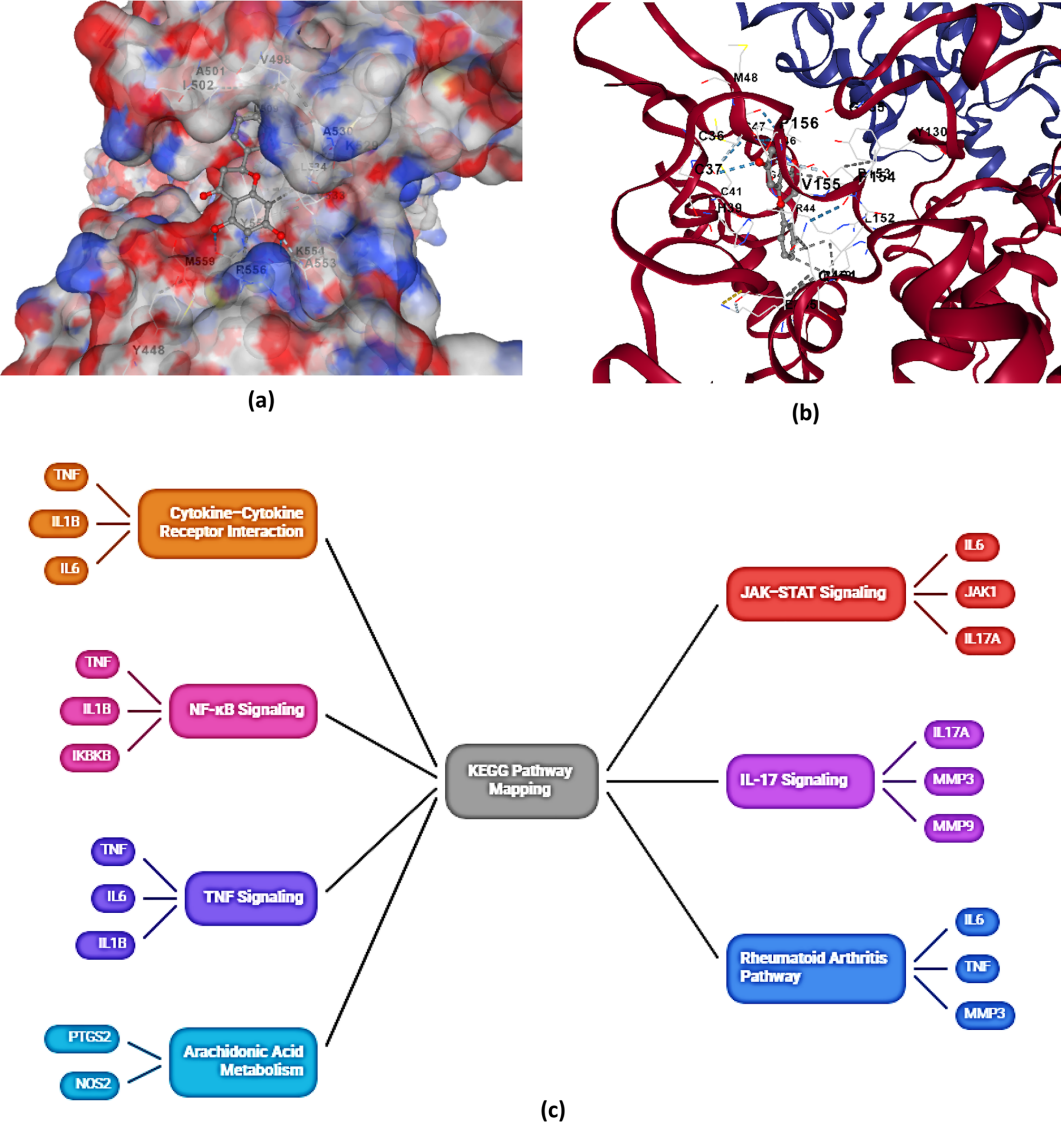

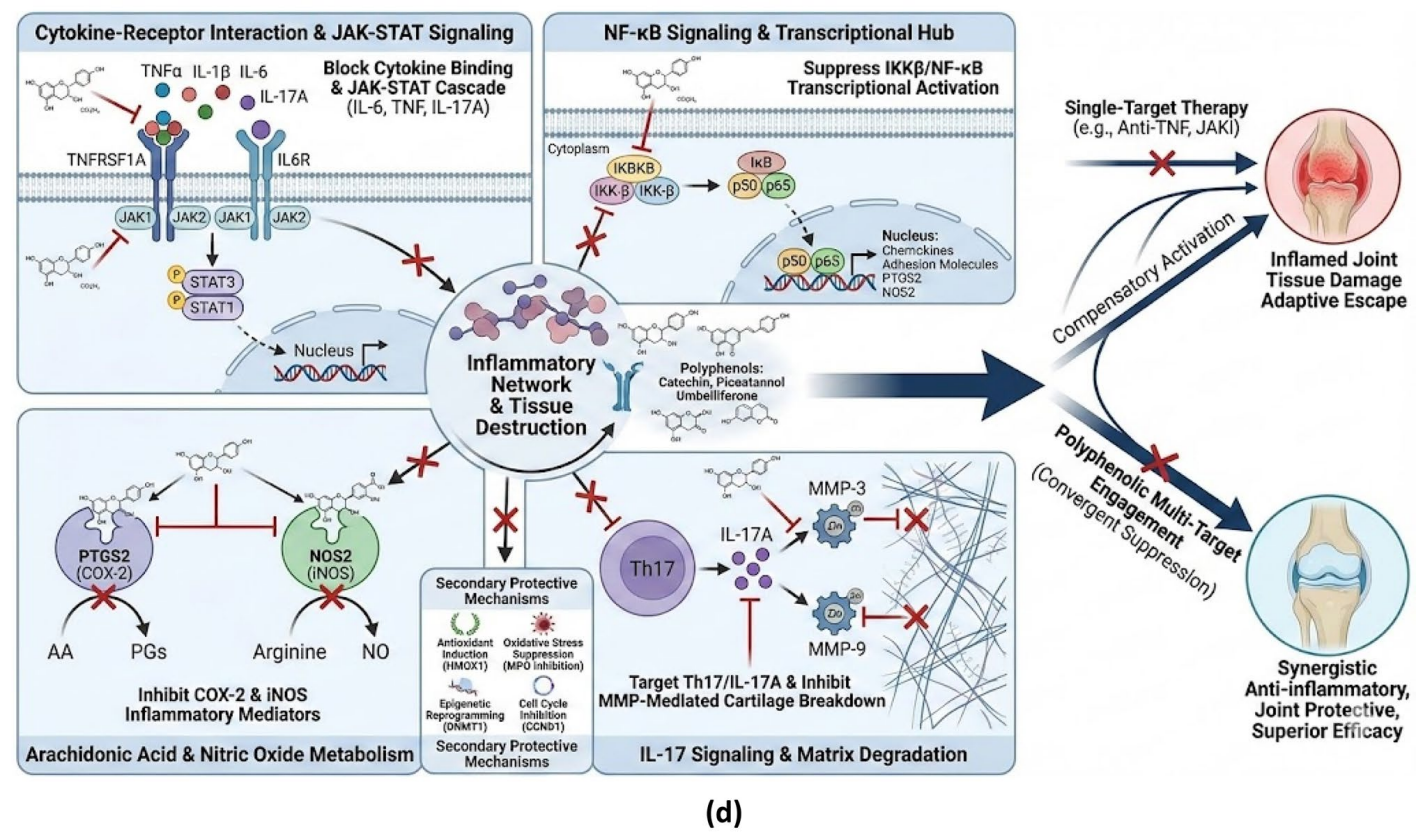
(**a**) Binding Pockets 7FCH: 68,071 (**b**) Binding Pockets 5F1A: 68,071 (**c**) KEGG pathway Diagram. (**d**) Multitargeting Pathways of Polyphenols

Docking analysis revealed that methotrexate consistently exhibited the highest binding affinities across pro-inflammatory cytokines (TNF-α − 9.0, IL-1β − 9.0, IL-6 − 7.4, IL-17 A − 9.5 kcal/mol), cytokine receptors (TNFR1 − 9.4, IL-6R − 10.4), acute phase protein CRP (− 8.7), matrix metalloproteinases (MMP-3 − 10.3, MMP-9 − 10.2 to − 10.3), signaling kinases (JAK1/JAK2 − 10.4, IKKβ − 10.3), and inflammatory enzymes (iNOS − 10.4, COX-2 − 10.6 to − 11.3), (Fig. 3(a-b)) representing selected binding pockets of the interaction.

Polyphenols Catechin, Piceatannol, Galangin, and Umbelliferone showed high to moderate affinities (− 6.1 to − 9.9 kcal/mol), targeting residues in receptor interfaces, catalytic sites, ATP-binding pockets, and heme/catalytic pockets, indicating potential inhibition of NF-κB, JAK-STAT, MMP-mediated matrix degradation, and NO-prostaglandin mediated inflammatory pathways, supporting anti-inflammatory and chondroprotective potential. Overall, docking revealed distinct binding patterns across ligands and targets. Methotrexate was consistently the strongest binder (average − 9.5 kcal/mol; range − 7.4 to − 11.3), followed by Catechin (− 8.7; −6.3 to − 10.3), Piceatannol (− 8.5; −6.2 to − 9.8), Umbelliferone (− 8.2; −6.0 to − 8.7), and Galangin (− 8.1; −5.0 to − 10.1), with Pinocembrin and Sinapic Acid showing moderate to low affinities. Target-specific trends highlighted iNOS as the most receptive, with Catechin matching methotrexate (− 10.3 kcal/mol), and COX-2 displaying strong polyphenol engagement (Catechin − 9.4, Piceatannol − 9.7 kcal/mol). MMP-9 interactions were robust across all polyphenols, supporting matrix-protective potential, whereas IL-6 showed the weakest affinities among cytokines, suggesting selective binding challenges. KEGG and GO enrichment linked the polyphenol-modulated target set to cytokine–cytokine receptor interaction, JAK–STAT, NF-κB, IL-17, TNF, RA, and arachidonic-acid pathways (Supplementary Table 3), as well as biological processes including inflammatory response, extracellular matrix degradation, and nitric-oxide biosynthesis (Supplementary Table 3), confirming that the ligand panel collectively covers the critical effector axes of RA pathogenesis, Fig. 3(c).

## Discussion

The integrated in silico analysis shows that five relatively unexplored polyphenols perform as pharmacokinetically superior, mechanistically broader RA modulators compared with methotrexate, and, in several dimensions, complement the phytoconstituent catechin. ADME profiling, docking, PPI/STITCH networks, and KEGG/GO shows a clear treatment pathway through this molecules attenuate cytokine signaling, kinase cascades, matrix degradation, and NO production, matching with current network-pharmacology concepts for RA, Fig. 3 (d).

Selected five test polyphenols (sinapic acid, galangin, piceatannol, umbelliferone and pinocembrin) and catechin satisfied Lipinski’s rule of five, with moderate lipophilicity (LogP approximately 0.8–2.3), high GI absorption, and bioavailability scores around 0.55, therefore genuinely drug-like oral profiles [17]. In contrast, methotrexate exhibited one Lipinski violation, extreme hydrophilicity (very low LogP), reduced skin permeability (logKp − 10.39 cm/s) and a bioavailability score of 0.11, indicating its known poor and variable oral exposure and need for parenteral dosing in clinical practice. Solubility models (ESOL, Ali, Silicos-IT) placed the polyphenols in a balanced solubility–permeability window, whereas methotrexate appeared as highly soluble but poor permeability, indicating its low GI absorption and strong P-gp behaviour [19]. CYP analysis favoured phytochemicals sinapic acid and catechin showed no predicted CYP inhibition, piceatannol and galangin inhibited a limited panel (CYP1A2 ± CYP2C9/CYP2D6/CYP3A4), and umbelliferone/pinocembrin selectively targeted CYP1A2, indicating isoform-restricted DDI risk compared with many small-molecule DMARDs [20].

Docking across 15 RA-relevant targets revealed a clear difference between methotrexate’s high-affinity, low-plasticity profile and the polyphenols’ distributed multi-target engagement. Methotrexate consistently produced the most negative binding energies (in between − 7.4 and − 11.3) across cytokines (TNF-α, IL-1β, IL-6, IL-17 A), receptors (TNFR1, IL-6R), kinases (JAK1/2, IKKβ), MMP-3/MMP-9 [21], and inflammatory enzymes (COX-2, iNOS), recognized antifolate potency [22]. Among polyphenols, catechin emerged as the benchmark, with average docking of − 8.7 kcal/mol and high-affinity binding at iNOS (− 10.3 kcal/mol, matching methotrexate) and COX-2 (− 9.4 kcal/mol) [23], while piceatannol (− 8.5 kcal/mol) and umbelliferone/galangin (− 8.1 to − 8.2 kcal/mol) also showed favourable scores against COX-2, MMP-3/MMP-9 and selected cytokine receptor. Sinapic acid and pinocembrin exhibited weaker but still meaningful affinities, suggesting roles as supportive rather than primary actives [24]. Importantly, MMP-9 and COX-2 binding was consistently strong across several polyphenols, highlighting a shared chondroprotective and analgesic potential that is absent from methotrexate’s direct mechanism [25].

STRING PPI analysis placed the 15 targets within a dense inflammatory backbone centred on TNF, IL1B, IL6, IL17A, TNFRSF1A, IL6R, JAK1/JAK2, IKBKB, MMP3/MMP9, PTGS2 and NOS2 nodes that integrate cytokine–cytokine receptor interaction, TNF, IL-17, JAK–STAT, NF-κB, rheumatoid arthritis (hsa05323) and arachidonic-acid pathways. High-confidence such as TNF–TNFRSF1A, IL6–IL6R, JAK1–JAK2, MMP3/MMP9–TIMP1 and PTGS2–NOS2 defined topological hubs whose modulation is predicted to yield disproportionate network impact, aligning with clinical experience that anti-TNF, anti-IL-6 and JAK inhibitors produce robust but incomplete responses [15, 21–23, 26]. STITCH mapping extended these insights by revealing that catechin and piceatannol strongly couple PTGS2 with antioxidant and immunoregulatory mediators (HMOX1, CSF2, DNMT1, PON1), while galangin connects CYP1B1 to MMP2, VCAM1 and MTOR, and umbelliferone links CYP2A6/UGTs to CCND1 and MPO, integrating drug metabolism, vascular inflammation, proliferative pannus formation and oxidative stress into the RA network. Pinocembrin showed a narrower interaction spectrum (AGT, SULT1A1/3), suggesting a more selective, low off-target. Methotrexate’s STITCH profile remained focused on DHFR–TYMS, confirming a single dominant antifolate axis [27].

KEGG enrichment corroborated that the combined target set populates the key RA effector pathways Cytokine–Cytokine Receptor Interaction (hsa04060), TNF (hsa04668), IL-17 (hsa04657), JAK–STAT (hsa04630), NF-κB (hsa04064), Rheumatoid Arthritis (hsa05323) and Arachidonic Acid Metabolism (hsa00590) while GO emphasised inflammatory response, cytokine-mediated signaling, regulation of NF-κB, positive regulation of JAK–STAT, extracellular matrix degradation and nitric-oxide biosynthesis as dominant biological processes [28]. Cytokines (TNF, IL1B, IL6, IL17A) mapped to cytokine activity and receptor binding; JAK1, JAK2 and IKBKB to kinase activity and cytoplasmic signaling; MMP3/MMP9 to metallopeptidase activity and ECM degradation; PTGS2/NOS2 to nitric-oxide biosynthetic and eicosanoid pathways; and all secreted mediators to the extracellular region or plasma membrane compartments [5, 27, 29].

In this study catechin is considered as reference polyphenol combining excellent ADME features, docking and dense STITCH connectivity, particularly around PTGS2–HMOX1–DNMT1–PON1 that shows inflammation, redox balance and epigenetic regulation. Piceatannol closely mirrors catechin’s performance at COX-2, MMP-9 and IL-17–linked nodes [30]. Galangin, with its flavonol core and planar C2–C3 double bond, shows relatively stronger links to CYP1 family enzymes, MTOR, MMP2 and VCAM1 used in modulating synovial angiogenesis and pannus growth. Umbelliferone’s interactions with CYP2A6, CCND1, and MPO, docked at COX-2 and MMPs indicates its dual anti-proliferative and anti-oxidative role in the synovium [31]. Pinocembrin shows selective engagement with AGT and sulfotransferases and moderate docking at COX-2/MMPs represent it with low off-target risk [26, 27]. Compared with catechin, these five phytoconstituents across metabolic enzymes, adhesion molecules, oxidative and proliferative regulators without losing the core cytokine JAK–NF-κB–MMP–PTGS2/NOS2 axis showing their potential in the management of RA [21, 24, 30]. Whereas methotrexate delivers stronger per-target affinity but operates through a narrow, proliferation-focused antifolate mechanism and lacking with from poor oral bioavailability and toxicity, whereas the polyphenols provides affinity at multiple effector nodes with better predicted safety and oral exposure [32].

## Conclusion

This study demonstrates that the selected polyphenols are orally suitable multi-target chemotype. These polyphenols target central RA inflammatory network by attenuating TNF, IL1B, IL6, IL17A and their receptors, inhibiting JAK1/JAK2 and IKBKB signalling, suppressing NF-κB and JAK–STAT transcription, and directly targeting MMP3/MMP9, COX-2 and iNOS to limit cartilage degradation and inflammatory mediator generation. Additional engagement of HMOX1, MPO, PON1, and DNMT1 indicates redox rebalancing and epigenetic modulation of inflammatory genes, and offering basis for sustained disease control rather than transient symptom relief.​ Overall ADME, docking, network, STITCH, KEGG and GO data supports treatment model in which five understudied phytoconstituents, particularly in combination with catechin, deliver convergent pathway suppression across cytokine, kinase, transcriptional, proteolytic and oxidative axes. This distributed inhibition is predicted to overcome compensatory pathway activation that limits anti-TNF and JAK inhibitor monotherapies, positioning these polyphenols as rational leads for next-generation, multi-target disease-modifying strategies in rheumatoid arthritis. However, experimental validation is required to confirm these predicted multi-target interactions, establish dose response relationships, and verifying true disease-modifying efficacy in vitro and in vivo models.

## Supplementary Information

Below is the link to the electronic supplementary material.

Supplementary Material 2

## Data Availability

No datasets were generated or analysed during the current study.
