# Supplementary material for: In silico analysis of selected polyphenols as potential multitarget rheumatoid arthritis modifying agents
Source: Mol Biol Rep. 2026 Jan 16;53(1):297. doi: 10.1007/s11033-026-11440-7 (PMC12811167; doi:10.1007/s11033-026-11440-7)
Supplement: Supplementary file 1 — Supplementary Material 2 [file 11033_2026_11440_MOESM1_ESM.docx]

**Supplementary files**

**List of Supplementary Tables**

**Supplementary Table 1.** Selected polyphenols and the Protein Targets for the study

| **Selected Polyphenol for the study.** | | | | | | | |
| --- | --- | --- | --- | --- | --- | --- | --- |
| 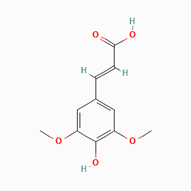 | | 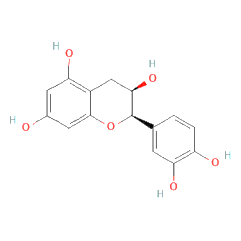 | | 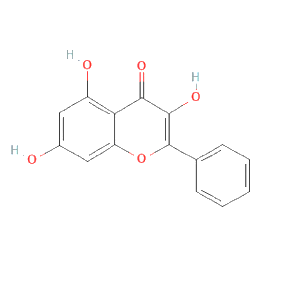 | | 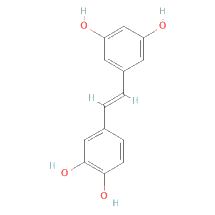 | |
| **Sinapic Acid**  **(637775)**  [**C11H12O5**](https://pubchem.ncbi.nlm.nih.gov/#query=C11H12O5) | | **Catechin**  **(72276)**  [**C15H14O6**](https://pubchem.ncbi.nlm.nih.gov/#query=C15H14O6) | | **Galangin**  **(5281616)**  [**C15H10O5**](https://pubchem.ncbi.nlm.nih.gov/#query=C15H10O5) | | **Piceatannol**  **(667639)**  [**C14H12O4**](https://pubchem.ncbi.nlm.nih.gov/#query=C14H12O4) | |
|  | | 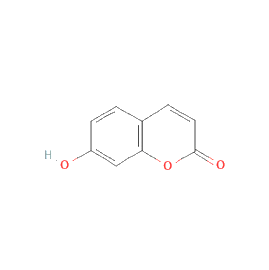 | | 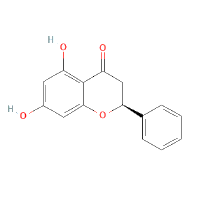 | |  | |
|  | | **(e) Umbelliferone (5281426) C_17_H_19_F_3_N_6_O** | | **(f) Pinocembrin**  **(68071)**  [**C15H12O4**](https://pubchem.ncbi.nlm.nih.gov/#query=C15H12O4) | |  | |
| **RA-associated protein targets.** | | | | | | | |
| **Pathway** | **Biomarker** | **Type** | **PDB ID** | **Pathway** | **Biomarker** | **Type** | **PDB ID** |
| **TNF Signaling** | TNF-α | Cytokine | 2AZ5 | **Acute Phase Response** | CRP | Protein | 1GNH, 1B09 |
|  | TNFR1 | Receptor | 1EXT | **Matrix Degradation** | MMP-3 | Enzyme | 2D1O, 1UEA |
|  |  |  |  |  | MMP-9 |  | 1GKC, 4H3X |
| **IL-Pathway** | IL-1β | Cytokine | 4DEP | **JAK-STAT Signaling** | JAK1 | Kinase | 3EYG |
|  | IL-6 |  | 1ALU |  | JAK2 |  | 2B7A |
|  | IL-17A |  | 4HSA | **NF-κB Pathway** | IKKβ |  | 4KIK |
|  | IL-18 |  | 2AZ5 | **Oxidative Stress** | iNOS | Enzyme | 1NSI, 3E7G |
|  | IL-6R | Receptor | 1N26 |  | COX-2 |  | 5F1A, 4COX |

**Supplementary Table 2.** Protein- Protein Interaction.

| **Protein A** | **Protein B** | **Combined Score** | **Protein A** | **Protein B** | **Combined Score** |
| --- | --- | --- | --- | --- | --- |
| **CRP** | IL6R | 0.725 | **IL1B** | PTGS2 | 0.974 |
|  | MMP9 | 0.806 |  | IL6 | 0.996 |
|  | IL1B | 0.886 |  | TNF | 0.998 |
|  | TNF | 0.938 | **IL6** | IL6R | 0.999 |
|  | IL6 | 0.968 |  | JAK1 | 0.996 |
| **IKBKB** | TNFRSF1A | 0.999 |  | JAK2 | 0.995 |
|  | TNF | 0.999 |  | TNF | 0.994 |
| **IL17RA** | IL1B | 0.774 |  | PTGS2 | 0.96 |
|  | IL6R | 0.71 |  | MMP9 | 0.959 |
|  | TNF | 0.825 |  | MMP3 | 0.887 |
|  | IL6 | 0.85 | **IL6R** | JAK1 | 0.986 |
| **IL1B** | TNFRSF1A | 0.997 |  | JAK2 | 0.963 |
|  | TIMP1 | 0.791 | **JAK1** | JAK2 | 0.999 |
|  | NOS2 | 0.803 | **MMP3** | TIMP1 | 0.999 |
|  | JAK2 | 0.806 | **MMP9** | TIMP1 | 0.999 |
|  | MMP3 | 0.832 | **NOS2** | PTGS2 | 0.908 |
|  | MMP9 | 0.949 |  |  |  |

**Supplementary Table 3.** Ligand Pathway Analysis: Stitch, KEGG, GO Analysis.

| **Ligand (ID)** | **Molecular Targets** | **Dominant KEGG Pathways** | **GO Biological Processes** | **Max Score** |
| --- | --- | --- | --- | --- |
| **Galangin (5281616)** | UGT1A7, UGT1A8, CYP1A1, GSTP1 | Xenobiotic metabolism, Arachidonic acid metabolism | Detoxification, oxidative stress regulation | 0.999 |
| **Umbelliferone** | CYP2A6, CYP2A13, UGT1A9 | Drug metabolism–CYP450 | Metabolic process, inflammatory modulation | 0.972 |
| **Catechin (72276)** | PTGS2, HMOX1 | Arachidonic acid metabolism, NF-κB signaling | Inflammatory response, antioxidant activity | 0.865 |
| **Pinocembrin (68071)** | AGT | Cytokine–cytokine receptor interaction | Regulation of inflammatory signaling | 0.786 |
| **Methotrexate (275651276)** | DHFR, TYMS | JAK–STAT signaling, Rheumatoid arthritis | Immune regulation, cytokine-mediated signaling | 0.998 |


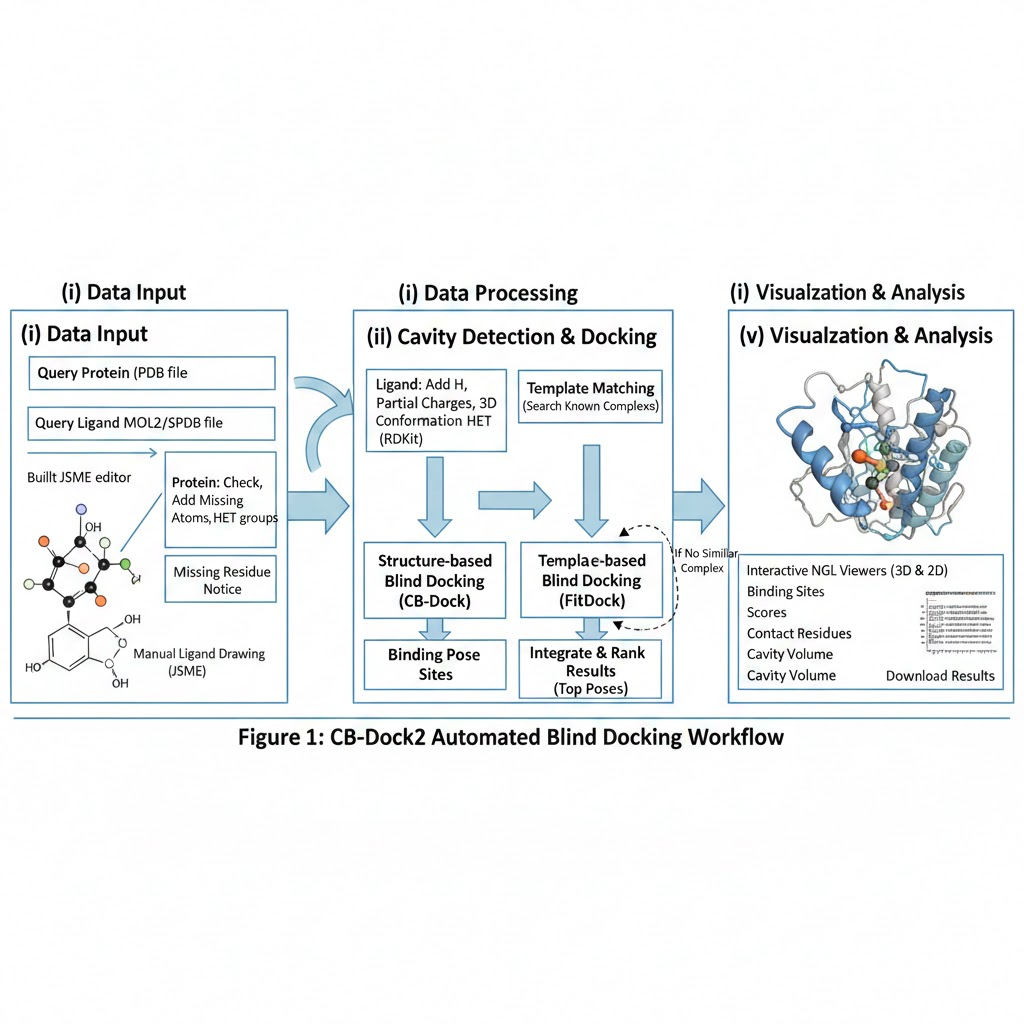


**Supplementary Figure 1.** CB Dock: Molecular Docking framework.
